# Patterns of self-medication in Qassim Province, Saudi Arabia: A cross-sectional study

**DOI:** 10.1016/j.amsu.2021.102207

**Published:** 2021-03-17

**Authors:** Fatimah AlGhofaili

**Affiliations:** Department of Dermatology, College of Medicine, Qassim University, King Abdulaziz Road, Almleda, 52571, Buraydah, Qassim, Saudi Arabia

**Keywords:** Drugs, Over the counter, Self-medication, Pharmacology, Prevalence

## Abstract

**Background:**

Selling medicines in pharmacies without a prescription is a common practice in the Kingdom of Saudi Arabia. The aim of this study was to determine the attitudes about and prevalence of non-prescribed medication for those attending community pharmacies in Qassim Province, Saudi Arabia.

**Methods:**

This is a cross-sectional survey based study that was designed and administered to individuals attending four community pharmacies in the main cites of Qassim Province, Saudi Arabia to obtain Over The Counter (OTC) drug use, exposure to health education, awareness of risks, important causes, and side-effects.

**Results:**

Approximately 75% of 109 individuals attending the studied pharmacies purchased non-prescribed medicines. Most participants (80%) had read the drug pamphlets and followed the instructions for use. The most common reasons for buying non-prescribed medicines were repetition of a previous prescription by a healthcare provider (30.3%), belief that the disease was minor (26.6%), convenience of pharmacist prescription (19.3%), and the low cost and shortness of time to attend health services. The most commonly used drugs were analgesics (18.3%) followed by anti-allergic medications and antibiotics, and the least commonly used drugs were laxatives and anti-diarrheal medications. There were no significant differences in the purchase of non-prescribed drugs with respect to age, gender, and educational level. Only 9% of respondents were opposed to buying non-prescribed drugs and 34% were willing to use them only when necessary.

**Conclusions:**

There was a high prevalence of non-prescribed medicine purchase from community pharmacies in the study area. Education efforts are needed to change this behavior.

## Highlights

• Self-prescribed medication can be harmful without medical knowledge.
• Antibiotic resistances have been previously linked to self-prescribed drugs.
• Explore which type of drugs are commonly self-prescribed in Qassim.

## 1 Introduction

Over the counter (OTC) drugs are estimated to be equal to or more than 100,000 drugs in the pharmaceutical markets [1,2]. The definition of these drugs includes any drugs that can be considered safe and efficacious for the public and can be administered with seeking medical consultation [3]. Self-medication is defined as the self-administration of certain drugs by the patient to manage a recognized illness or symptom with no previous medical experience according to the World Health Organization [4]. Moreover, it has now become obvious that more and more drugs are being available as OTC for the public. Self-medication and frequent consumption of non-prescribed medications can lead to many serious complications. The commonest and most serious complication is the potential occurrence of adverse drug reactions (ARDs) between the administered medications [5]. Previous studies showed that increased intake of multiple drugs increases the risk of developing ARDs [5,6].

The risk is also higher in older patients as evidence shows that the frequency of administration of non-prescribed drugs can increase up to seven times more than that of prescribed ones [7,8]. Older patients might also suffer from a renal and hepatic impairment, increased body fat, and deterioration of an underlying medical condition [2,9]. Besides, around one-third of adults’ hospital admissions are correlated with non-prescribed drug administration [5]. Besides, the risk for having a side effect is even higher due to the increased frequency of drug administration. Such side effects include drug-to-drug and drug-to-food interactions, allergic reactions, drug tolerance, and masking of other clinical symptoms that may underly a serious clinical condition. Thus, further efforts should be applied to face this phenomenon and raise public awareness of the potential side effects [10].

Despite the presence of adequate regulations for OTC consumption in Saudi Arabia, evidence shows that prescribed medications are commonly purchased without a prescription. Aljadhey et al. [11] reported that around 22% of antibiotics are being bought with no prescriptions. Evidence shows that the habit of buying and selling non-prescribed drugs is common, according to previous reports from Saudi Arabia [12,13]. However, no previous reports from the kingdom have focused whether on obtaining a specific rate or annual sales of the non-prescribed drugs. Consequently, the present study aims to investigate the patterns of OTC medicine use in the general population of Qassim, Saudi Arabia. We will also report the magnitude of OTC use, the commonest purchased drugs and the reason for this purchase, the presence of secondary side effects, and the public awareness towards this issue. By the end of this study, we aim to identify areas that can be targeted by health education campaigns to reduce the prevalence of OTC drug abuse.

## 2 Methods

This was a cross-sectional study of 109 patients attending four community pharmacies in Qassim, Saudi Arabia. Data were collected using a specially designed survey. The survey was built based on the data obtained from previous similar studies, globally and within Saudi Arabia. The contents of the survey and the study objectives were explained to the pharmacists, who provided informed consent. Pharmacists were also asked to educate patients, that agreed to participate in the survey, about the aim of the study and the possible outcomes and implications. Besides, they were asked to give the patients a brief about self-medication. All personal information regarding the study participants was kept anonymus.

The questionnaire was distributed to the pharmacists, who gave it to the patients to complete on their own. We aimed to target all types of patietns who were able to complete the questionnaire and answer the related questions to collect data from all potential ages and community classes. The language of the questionnaire was also written in Arabic and English for better comprehension of any potential patients visiting the pharmacy. The completed surveys were collected from the pharmacies two months later. The pharmacies and individuals visiting the pharmacies provided informed consent. The collected data include patients’ demographics as age, gender, and level of education. Our questionnaire was also designed to collect data regarding the personal and medical history, with specific questions asked about OTC drugs, exposure to health education, awareness of risks, important causes, and side effects from our targeted population.

Data were entered into and analyzed using SPSS for Windows v26 (IBM Statistics, Armonk, NY). Variables were presented as counts and percentages, with performing logictic regression to test the association of differenr predictors with the usage of OTC drugs. P-value<0.05 was considered significant. This study was written with STROCSS 2019 guideline of reporting [14].

## 3 Results

**Fig. 1:**
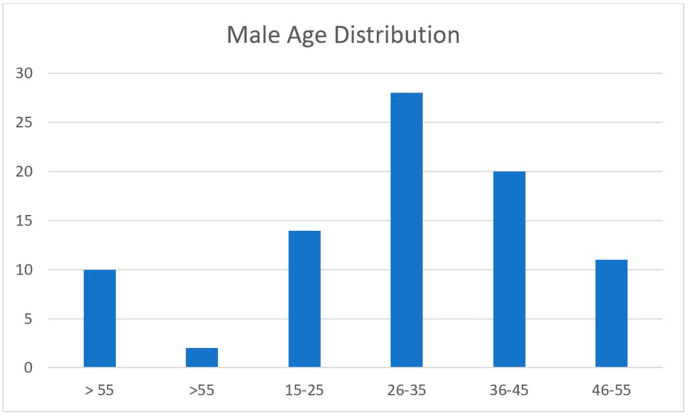
Distribution of male participants according to age.

**Fig. 2:**
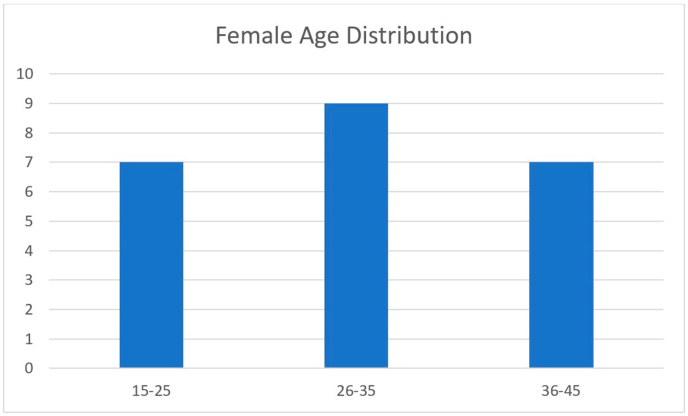
Distribution of Female participants according to age.

Table 1 shows the baseline characteristics of the participants. Nearly 80% of participants were male and about 80% were aged under 45 years Fig. 1, Fig. 2. Approximately 75% of participants in the studied pharmacies were purchasers of OTC medicines, with no prescriptions. Most participants (80%) had read the drug-associated pamphlets and followed their instructions for use. Only 9% of respondents were opposed to buying non-prescription drugs, and 34% agreed to use OTC only when necessary. Noteworthy, only 7.3% of the participants reported encountering side effects (Table 2). According to the logistic regression results, there were no significant associations between the purchase of non-prescription drugs and age, gender, and educational level (Table 3).

**Table 1:** Baseline characteristics of the participants.

Variable	Count	%
Age		
15-25	21	19.2
26-35	37	33.9
36-45	27	24.8
46-55	11	10.1
>55	13	11.9
**Gender**		
Female	23	21.1
Male	86	78.9
**Educational level**		
Primary	4	3.7
Intermediate	22	20.2
Secondary	41	37.6
University	35	32.1
Illiterate	7	6.4

**Table 2:** The OTC drug use by the participants.

Variable	Count	%
Drug with prescription		
Yes	27	24.8
No	82	75.2
**Read drug instructions**		
Read & followed	87	79.8
Read but did not follow	12	11.0
Did not read	10	9.2
**Approval of OTC drug use**		
Yes	62	56.9
No	10	9.2
When necessary	37	33.9
**Side-effects**		
Yes	8	7.3
No	101	92.7

**Table 3:** Logistic regression of different predictors to use OTC.

Predictor	Estimate	SE	Z	Odds ratio	95% Confidence Interval		P-value
					Lower	Upper	
Age							
15-25	*Reference*						
26-35	0.37	0.63	0.59	1.45	0.42	4.95	0.553
36-45	−0.39	0.63	−0.62	0.68	0.2	2.32	0.538
46-55	0.59	0.92	0.64	1.80	0.3	10.9	0.522
>55	1.39	1.15	1.2	4.00	0.42	38.45	0.23
**Gender**							
Female	*Reference*						
Male	0.09	0.54	0.16	1.09	0.38	3.13	0.869
**Education**							
Illiterate	*Reference*						
Primary	−1.79	1.47	−1.22	0.17	0.01	2.98	0.224
University	−0.22	1.17	−0.18	0.81	0.08	7.97	0.853
intermediate	−1.03	1.17	−0.88	0.36	0.04	3.56	0.38
secondary	−0.79	1.14	−0.69	0.45	0.05	4.21	0.488

SE: standard error.

**Figure 3:**
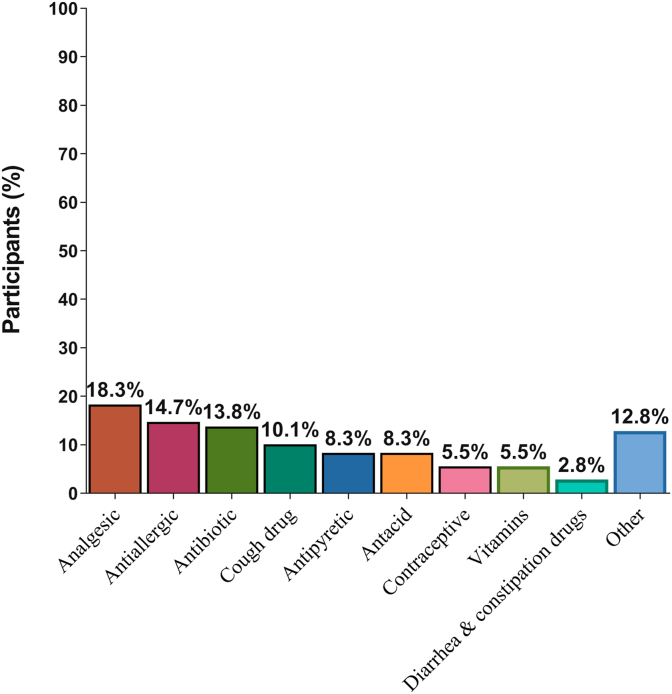
Types of OTC drugs bought by the participants (%)

**Figure 4:**
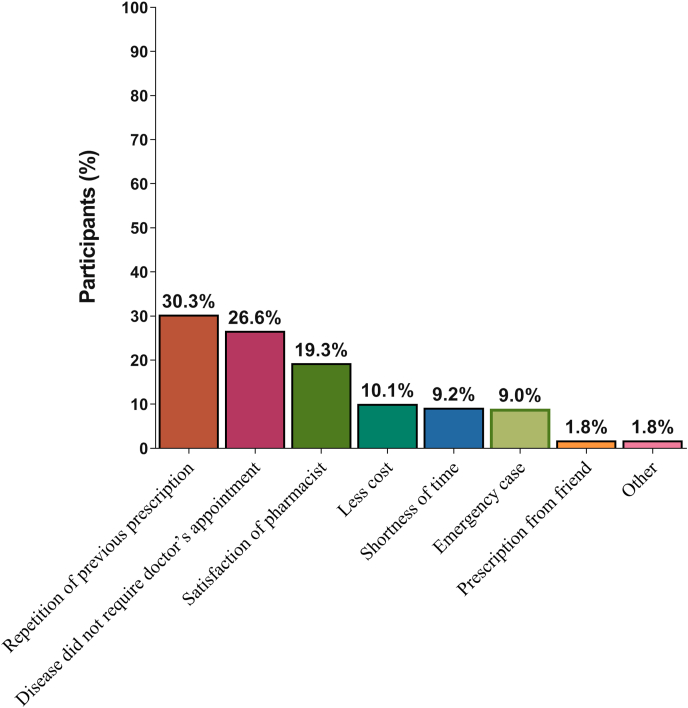
Reason for use of OTC drugs by the participants (%)

The most common reasons for buying OTC medicines were repetition of previous prescriptions (30.3%), treating a simple disease (26.6%), convenience of a pharmacist prescription (19.3%), and the low cost and ease of attending health services. Additionally, there were very few emergency cases (9%) (Fig. 3). In the same cotext, the most common drugs purchased were analgesics (18.3%) followed by anti-allergic drugs and antibiotics, with laxatives and anti-diarrheal drugs the least common drugs purchased (Fig. 4).

## 4 Discussion

This study highlights the problem of self-medication use in Qassim Province, Saudi Arabia. The prevalence of self-medication without a prescription was 75.2% while only 9.2% of participants opposed the use of OTC drugs. Our rate is similar to the 72.5% rate that was reported by Mahrous et al. [15] in Al Madina city and higher than the one reported by Aldeeri et al. [16] in Riadh, being 63.5%. On the other hand, our prevalence rate is a little less than the rate reported by Al-Ghamdi et al. [17], who conducted a similar survey from participants across the Kingdom and found that 81.3% out of 1630 participants sought self-medication with no prescriptions from healthcare professionals. Another cross-sectional study from Majmaah city also reported that 93.1% of the study participants used non-prescribed drugs [18]. These rates indicate the high prevalence of seeking non-prescribed self-medication practices among patients within Saudi Arabia which necessitates that further efforts should be applied to increase the community awareness about the potential hazards of the frequent consumption of OTC drugs.

Our results also show that the most commonly purchased drugs include analgesics, antiallergics, and antibiotics. Another recent study in Saudi Arabia showed that analgesics and antipyretics were the most commonly self-administered drugs followed by cough syrup, eye drops, and antibiotics [17]. A previous report from Saudi Arabia showed that analgesics and antibiotics were the most commonly used drugs in the Kingdom [19]. The study by Aldeeri et al. [16] reported that salt in hot water and acetamenophin were the most commonly used drugs. Besides, the previous study by Aljadhey et al. [11] reported that among 285 medications that were purchased without a prescription, only 51.2% were from OTC drugs while the others were drugs that require prescriptions. The authors also reported that only 29.2% of the study participants were aware of the potential hazards of purchasing non-prescribed medications. These findings indicate the wrong beliefs about non-prescribed drug self-administration and the need for greater supervision and proper guidelines of pharmacies [20].

We also found that the most common reasons for self-medication in this study include repetition of the previous prescription and that the disease did not require a prescription from a healthcare professional. Aljadhey et al. [11] reported that the commonest reasons included too minor symptoms to consult a physician and time-saving. The latter reason was also the commonest reason reported by Aldeeri et al. [16]. Moreover, Al-Ghamdi et al. [17] reported that the commonest reasons were the difficulties that patients had to face to reach a hospital, poor medical care, and lack of insurance or increased costs for medical consultation. Moreover, most of our study participants were either secondary school or university students. This is consistent with the previous results by Saeed et al. [21], who showed that 86.6% of the university students in Al-Qassim Province reported self-medication practices. Although we did not find any significant correlation between educational levels and drug self-administration, Al-Ghamdi et al. [17] reported that self-medication was more common among participants with a university-level education. No statistical significance was also found between gender or age and self-medication unlike previous studies which showed that self-medication was correlated more with females and patients at 20–29 years of age [17,22,23].

Apart from the reasons that patients used to justify self-medication, other reasons can also be considered. These include the potential competition between pharmacies which is attributable to the commercial pressure from drug companies who may offer discounts to pharmacists purchasing large quantities of their products. This indicates the role of pharmacists in reducing such high prevalence rates of self-medication by dealing with high professionalism to raise public awareness. This can be achieved by putting more strict guidelines on non-prescribed medications and educating pharmacists about OTC drugs as defined by the Ministry of Health in Saudi Arabia which also requires an urgent update. Moreover, the interpretation and current practice of community pharmacy needs to be changed to provide a better service to the public.

The current study was limited by the study design as data was obtained by self-reporting, and the sample size was relatively small as many pharmacists refused to participate in the study, so the results may not be representative.

## 5 Conclusion

In conclusion, there is a high prevalence of OTC drug use in Qassim Province. Analgesics, antiallergics, and antibiotics are the most commonly purchased drugs with no prescriptions. Consequently, these results indicate the need to increase community awareness about the dangers of using OTC drugs by the further inauguration of health education campaigns.

## Annals of medicine and surgery

Please state any conflicts of interest.

## Ethical approval

IRB Approval was obtained by King Fahad Armed Forces Hospital in Jeddah, KSA.

## Consent

All patients were informed over the phone by the IRB that they will be included in the study with their data being private and protected.

## Author contribution

All authors contributed evenly in drafting, collecting data, analysis, writing and proofreading of the paper.

## Registration of Research Studies

In accordance with the Declaration of Helsinki 2013, all research involving human participants has to be registered in a publicly accessible database. Please enter the name of the registry and the unique identifying number (UIN) of your study.

You can register any type of research at http://www.researchregistry.com to obtain your UIN if you have not already registered. This is mandatory for human studies only. Trials and certain observational research can also be registered elsewhere such as: ClinicalTrials.gov or ISRCTN or numerous other registries.

1. Name of the registry: Research Registry
2. Unique Identifying number or registration ID: researchregistry6565
3. Hyperlink to your specific registration (must be publicly accessible and will be checked): https://www.researchregistry.com/browse-the-registry#user-researchregistry/registerresearchdetails/60267f4d175c2b001b5384fc/

## Guarantor

Ashwag Alwagdani

## Funding

None.

## Declaration of competing interest

The author declares no conflict of interest.

## References

[1] Fakeye T.O., Adisa R., Olatunji E. (2010). Self medication among hospitalized patients in selected secondary health facilities in South Western Nigeria. Pharm. Pract..

[2] Rolita L., Freedman M. (2008). Over-the-counter medication use in older adults. J. Gerontol. Nurs..

[3] Administration USFaD Drug applications for over-the-counter (OTC) drugs 2020. https://www.fda.gov/drugs/types-applications/drug-applications-over-counter-otc-drugs.

[4] World Health O (1998). The Role of the Pharmacist in Self-Care and Self-Medication : Report of the 4th WHO Consultative Group on the Role of the Pharmacist, the Hague, The Netherlands, 26-28 August 1998.

[5] Curry L.C., Walker C., Hogstel M.O., Burns P. (2005). Teaching older adults to self-manage medications: preventing adverse drug reactions. J. Gerontol. Nurs..

[6] Golden A.G., Preston R.A., Barnett S.D., Llorente M., Hamdan K., Silverman M.A. (1999). Inappropriate medication prescribing in homebound older adults. J. Am. Geriatr. Soc..

[7] Conn V.S. (1992). Self-management of over-the-counter medications by older adults. Publ. Health Nurs..

[8] Amoako E.P., Richardson-Campbell L., Kennedy-Malone L. (2003). Self-medication with over-the-counter drugs among elderly adults. J. Gerontol. Nurs..

[9] Francis S.A., Barnett N., Denham M. (2005). Switching of prescription drugs to over-the-counter status: is it a good thing for the elderly?. Drugs & aging.

[10] Abdi A., Faraji A., Dehghan F., Khatony A. (2018). Prevalence of self-medication practice among health sciences students in Kermanshah, Iran. BMC. Pharmacol. Toxicol..

[11] Aljadhey H., Assiri G.A., Mahmoud M.A., Al-Aqeel S., Murray M. (2015). Self-medication in Central Saudi Arabia. Community pharmacy consumers' perspectives. Saudi Med. J..

[12] Al-Mohamadi A., Badr A., Bin Mahfouz L., Samargandi D., Al Ahdal A. (2013). Dispensing medications without prescription at Saudi community pharmacy: extent and perception. Saudi Pharmaceut. J. : SPJ: Off. Publ.Saudi.Pharm. Soc.

[13] Bin Abdulhak A.A., Altannir M.A., Almansor M.A., Almohaya M.S., Onazi A.S., Marei M.A. (2011). Non prescribed sale of antibiotics in Riyadh, Saudi Arabia: a cross sectional study. BMC Publ. Health.

[14] Agha R., Abdall-Razak A., Crossley E., Dowlut N., Iosifidis C., Mathew G. (2019). STROCSS 2019 Guideline: strengthening the reporting of cohort studies in surgery. Int. J. Surg..

[15] Mahrous M.S. (2018). Frequency of use of non-prescribed medication among population sample from Al Madina City and its impact on quality of care in Saudi Arabia. Int. J. Health Sci..

[16] Aldeeri A., Alzaid H., Alshunaiber R., Meaigel S., Shaheen N.A., Adlan A. (2018). Patterns of self-medication behavior for oral health problems among adults living in riyadh, Saudi Arabia. Pharmacy.

[17] Al-Ghamdi S., Alfauri T.M., Alharbi M.A., Alsaihati M.M., Alshaykh M.M., Alharbi A.A. (2020). Current self-medication practices in the Kingdom of Saudi Arabia: an observational study. Pan Afr Med J.

[18] Alzahrani M., Alhindi T., Almutairi A., Aldajani M., Sami W. (2015). Frequency of using non-prescribed medication in Majmaah city, Saudi Arabia -- A cross sectional study. JPMA.J. Pak. Med. Assoc.

[19] AlKhamees O.A., AlNemer K.A., Bin Maneea M.W., AlSugair F.A., AlEnizi B.H., Alharf A.A. (2018). Top 10 most used drugs in the Kingdom of Saudi Arabia 2010-2015. Saudi Pharmaceut. J. : SPJ: Off. Publ.Saudi.Pharm. Soc.

[20] Khan T.M. (2014). The consequences of nonprescription medication sales in Saudi Arabia's community pharmacies: regulations without implementation. Therapeutic advances in drug safety.

[21] Saeed M., Alkhoshaiban A., Al-Worafi Y., Ming L.C. (2014). Perception of self-medication among university students in Saudi Arabia. Arch. Pharm. Pract..

[22] Moraes A., Delaporte T., Fernandes C., Mario F. (2011). Factors associated with medicine use and self medication are different in adolescents. Clinics (São Paulo, Brazil).

[23] Jerez-Roig J., Medeiros L.F., Silva V.A., Bezerra C.L., Cavalcante L.A., Piuvezam G. (2014). Prevalence of self-medication and associated factors in an elderly population: a systematic review. Drugs & aging.

